# Structural Insights into Human Adenovirus Type 4 Virus-Associated RNA I

**DOI:** 10.3390/ijms23063103

**Published:** 2022-03-13

**Authors:** Helen Bergquist, Raviteja Inturi, Rula Zain, Tanel Punga

**Affiliations:** 1 Department of Medical Biochemistry and Microbiology, Uppsala University, BMC Box 582, Husargatan 3, SE-751 23 Uppsala, Sweden; hebe7619@gmail.com (H.B.); raviteja.inturi@imbim.uu.se (R.I.); 2 Department of Laboratory Medicine, Translational Research Center Karolinska (TRACK), Karolinska Institutet, Huddinge, SE-141 86 Stockholm, Sweden; rula.zain@ki.se; 3 Centre for Rare Diseases, Karolinska University Hospital, SE-171 76 Stockholm, Sweden

**Keywords:** adenovirus, VA RNAI, triplex RNA, BQQ–OP

## Abstract

RNA molecules can adopt specific RNA triplex structures to execute critical biological functions. Human adenoviruses (HAdVs) are abundant pathogens encoding the essential, noncoding virus-associated RNA I (VA RNAI). Here, we employ a triplex-specific probing assay, based on the intercalating and cleaving agent benzoquinoquinoxaline 1, 10-phenanthroline (BQQ–OP), to unravel a potential RNA triplex formation in VA RNAI. The BQQ–OP cleavage of the pathogenic HAdV type 4 (HAdV-4) VA RNAI indicates that a potential triplex is formed involving the highly conserved stem 4 of the central domain and side stem 7. Further, the integrity of the HAdV-4 VA RNAI side stem 7 contributes to a potential triplex formation in vitro and virus growth in vivo. Collectively, we propose that the HAdV-4 VA RNAI can potentially form a biologically relevant triplex structure.

## 1. Introduction

RNA molecules play an important role in regulating a variety of cellular processes including, but not limited to, chromatin modifications, pre-mRNA processing, and protein translation [1]. In order to carry out these critical functions, the RNA molecules often adopt specific three-dimensional structures. The three-dimensional structure is promoted by the maximization of base stacking, electrostatic stabilizations, and hydrophobic interactions [2]. Further, the tertiary structure is stabilized through the formation of different types of hydrogen bonds, including minor groove interactions, Watson–Crick, Hoogsteen, and reverse Hoogsteen base-pairing leading to tetraloops and triple-strand structures [3]. The triplex structures form when a third, single-stranded nucleotide chain binds in a sequence-specific manner to a polypurine/polypyrimidine duplex. The nucleobases of the third strand can either be purines or pyrimidines, and they bind to the polypurine stretch of the duplex via Hoogsteen or reverse Hoogsteen hydrogen bonds [4]. The stability of a given triplex structure is dependent on the sequence and on the surrounding environment, including the salt concentration, pH, and triplex-interacting proteins [5,6].

In order to study biologically relevant triplex structures, the low molecular weight molecule, benzoquinoquinoxaline (BQQ), was designed to specifically intercalate into and stabilize DNA triplex structures of both purine and pyrimidine motifs [7,8,9,10]. A follow-up study indicated that BQQ does not induce the formation of a triplex structure but is rather intercalated into a pre-formed structure [11]. Furthermore, BQQ was converted to a triplex-specific cleaving agent (BQQ–OP) by chemical conjugation to a 1, 10-phenanthroline (OP) derivative [8]. In the presence of Cu^2+^ ions and a reducing agent, the OP moiety of BQQ–OP produces in situ radicals, which cause the double-strand cleavage of nucleic acids, specifically at the site of triplex formation. Notably, BQQ–OP has previously been used to probe for triplex formation in DNA, which is formed either in an inter- or intramolecular manner (H-DNA) in plasmids and in shorter DNA fragments in vitro [11,12,13,14]. However, BQQ–OP has, until now, never been tested in probing an RNA structure.

Human adenoviruses (HAdVs) are common pathogens, causing a broad spectrum of diseases [15,16]. Remarkably, HAdV types 4 and 7 (HAdV-4 and HAdV-7) can cause outbreaks of acute respiratory illnesses among military recruits and children [17,18]. A particular feature of HAdVs is that they encode highly abundant, non-coding virus-associated RNAs (VA RNAI and VA RNAII) [19,20]. Although more than 110 HAdV types have been described (http://hadvwg.gmu.edu/ (accessed on 8 March 2022)), the majority of VA RNA studies are based on the highly similar HAdV-2 or HAdV-5 types (reviewed in [21,22]). The VA RNAI gene is essential, as its deletion drastically decreases the HAdV-5 virus titer; meanwhile the lack of VA RNAII does not show a similar effect on virus growth [23,24]. VA RNAI has multiple functions, including translation regulation, microRNA processing, and immune response modulation during lytic HAdV infection [22]. For example, VA RNAI can inhibit the anti-viral dsRNA-activated kinase PKR to keep the translation initiation factor eIF2α in unphosphorylated form, which allows efficient synthesis of the virus capsid proteins [25,26,27].

VA RNAI has a multipart secondary structure with three separate domains: the apical stem, central domain, and terminal stem (reviewed in [21,22]). In addition, individual short stem structures and internal bulge loops have been characterized on VA RNAI [20,28]. There are three main VA RNA sequence elements conserved between different HAdV types [19,28]. These elements are box A and box B, present in the terminal and apical stem, a complementary tetranucleotide sequence GGGU-ACCC present in the central domain, and a transcription terminator sequence at the 3′ end of the molecule. Although the VA RNAI secondary structure seems to be conserved, the VA RNAs originating from different HAdV types display considerable variations in their nucleotide sequence and length [19,28]. Hence, it is possible that HAdV type-specific VA RNAs possess particular intramolecular interactions, which would favour the formation of distinctive tertiary structures essential for VA RNAI functions. Indeed, it has been shown that HAdV type-specific VA RNAs are processed differently by the Dicer enzyme and that the expression of the HAdV type-specific VA RNAI affects eIF2α phosphorylation diversely [25,29].

In order to unravel the potential tertiary structure formation in VA RNAI, we tested BQQ–OP as a potential novel RNA triplex probing chemical. Our results show that HAdV type-specific VA RNAIs react differently to the BQQ–OP treatment. Furthermore, we demonstrated that BQQ–OP cleaves the HAdV-4 VA RNAI at the conserved 5′-GGGU-3′ motif and that this particular cleavage is influenced by the sequence integrity at the conserved stem 4 of the central domain and protruding stem 7.

## 2. Results

### 2.1. Differential Cleavage of HAdV-4, HAdV-5, HAdV-12, and HAdV-37 VA RNAI by BQQ–OP

**Figure 1:**
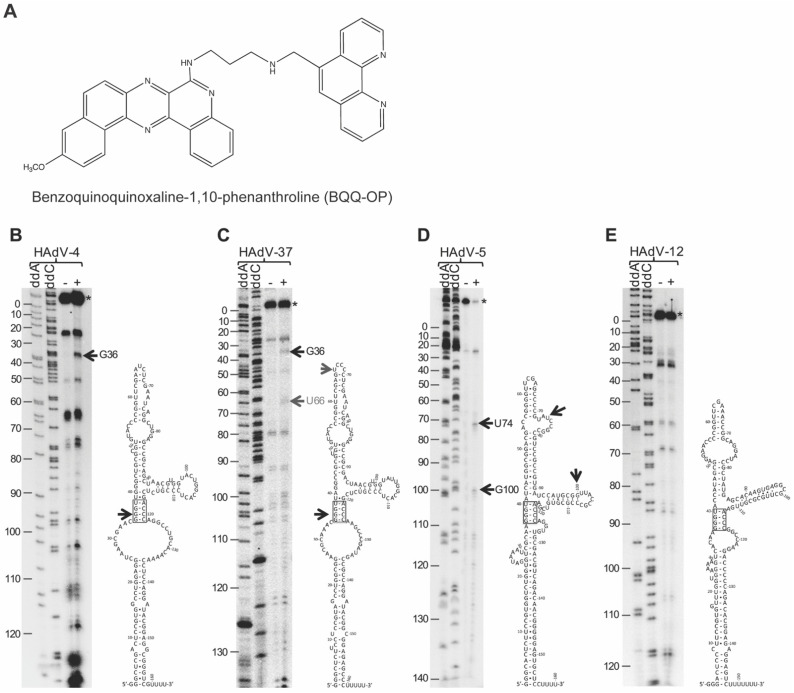
Differential cleavage of HAdV-4, HAdV-37, HAdV-5, and HAdV-12 VA RNAI by BQQ–OP. (**A**) Structure of the benzoquinoquinoaxline 1, 10-phenanthroline (BQQ–OP). Differential cleavage of ^32^P-labelled VA RNAI from HAdV-4 (**B**), HAdV-37 (**C**), HAdV-5 (**D**), and HAdV-12 (**E**) by BQQ–OP. The schematic secondary structure models of HAdV-4, HAdV-12, and HAdV-37 VA RNAI were derived from [28], whereas the schematic structure of HAdV-5 is from [33]. The hyphen (-) indicates incubation without BQQ–OP, whereas the plus (+) shows incubation in the presence of BQQ–OP. The horizontal arrows point towards specific VA RNAI cleavage products. The asterisk (*) marks the full-length, uncleaved VA RNAI. Sanger sequencing reactions with dideoxynucleotides (ddC and ddA) on the HAdV-4, HAdV-37, HAdV-5, and HAdV-12 VA RNAI containing plasmids were used for VA RNAI nucleotide numbering from 5′ to 3′.

We have previously shown that different VA RNAI molecules originating from the HAdV-4, HAdV-5, HAdV-12, and HAdV-37 do not equally well rescue the VA RNAI-deficient HAdV-5 (dl705) virus growth [25]. One potential explanation for the impaired rescue could be that the type-specific VA RNAIs fold into different higher-order structures, which affect their corresponding binding affinity and activity. Notably, our previous studies have established that BQQ–OP (Figure 1A) can be used to probe DNA triplex structures in vitro by monitoring a specific double-strand DNA cleavage at the site of the triplex formation [8,11,12,13,14]. Inspired by these findings and considering that VA RNAIs have been suggested to adopt the complex tertiary structures [30,31,32], we decided to test if the VA RNAI can form higher order structures, such as a triplex. For this purpose, we took advantage of our established BQQ–OP cleavage assay on triplex DNA substrates [11,13] and examined whether the BQQ–OP can also cleave various HAdV type-specific VA RNAIs. As shown in Figure 1B, BQQ–OP, in the presence of Cu^2+^ and a reducing agent, caused the specific cleavage of the HAdV-4 VA RNAI at nucleotide G36, located in the middle of the tetranucleotide 5′-GGGU-3′ motif, which forms a so-called stem 4 structure [20]. A similar cleavage pattern, although less prominent, was observed in the case of the HAdV-37 VA RNAI (Figure 1C), with an extra cleavage event at uracil 66 (U66). Additional VA RNAI cleavage products were observed both in the presence and absence of the BQQ–OP treatment, indicating that these are not triplex-specific cleavage events but, rather regions of the VA RNAI that are susceptible to hydrolysis under our experimental conditions. The closer inspection of the cleavage products indicated that they correspond to single-strand regions in the proposed secondary structures [19,28]. Interestingly, the HAdV-5 and HAdV-12 VA RNAI did not show the same cleavage patterns as were observed for the HAdV-4 and HAdV-37 VA RNAI. In the case of the HAdV-5 VA RNAI, two possible cleavage sites at guanosine 100 (G100) and uracil 74 (U74) were detected (Figure 1D). This observation implies that a triplex structure may form in the HAdV-5 VA RNAI; however, this structure is not the same as seen in the HAdV-4 and HAdV-37 VA RNAI. In contrast, the HAdV-12 VA RNAI was resistant to the BQQ–OP cleavage under the same experimental conditions (Figure 1E).

### 2.2. BQQ–OP Cleavage Specificity of the HAdV-4 VA-RNAI

**Figure 2:**
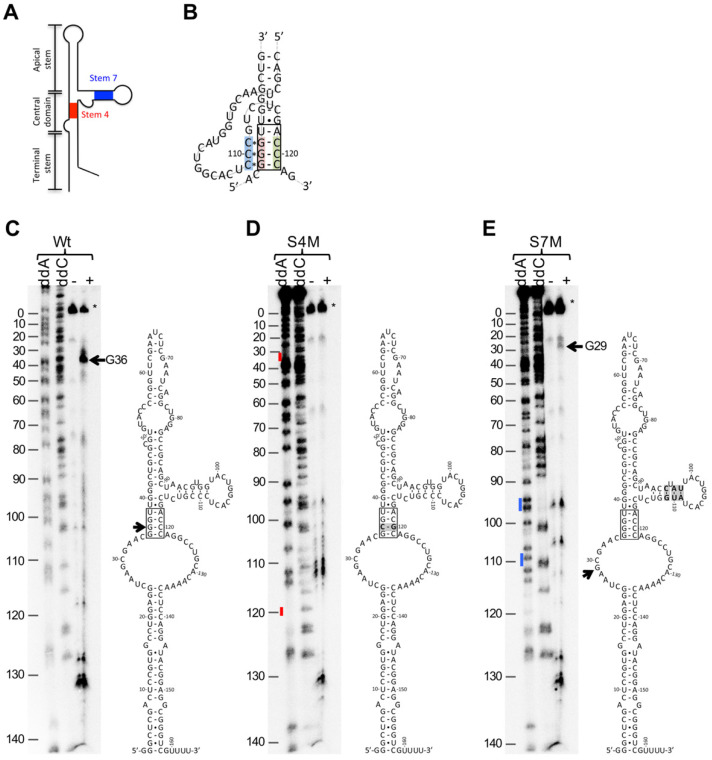
BQQ–OP cleavage specificity of the HAdV-4 VA-RNAI. (**A**) Schematic illustration of the HAdV VA RNAI structure. (**B**) Schematic illustration of a potential HAdV-4 VA RNAI triplex formation between the stem 4 and stem 7 sequences. Dashes (-) represent Watson–Crick base-pairing, whereas asterisks (*) represent Hoogsteen base-pairing. (**C**) BQQ–OP cleavage pattern of HAdV-4 VA RNAI (Wt). (**D**) BQQ–OP cleavage pattern of HAdV-4 VA RNAI (S4M). (**E**) BQQ–OP cleavage pattern of HAdV-4 VA RNAI (S7M). The hyphen (-) indicates incubation in the absence of BQQ–OP, whereas the plus (+) shows incubation in the presence of BQQ–OP. Horizontal arrows point towards specific VA RNAI cleavage products. The asterisk (*) marks the full-length, uncleaved VA RNAI. Sanger sequencing reactions with dideoxynucleotides (ddC and ddA) on HAdV-4 VA RNAI (Wt, S4M, S7M) containing plasmids were used for VA RNAI nucleotide numbering from 5′ to 3′. Stem 4 and stem 7 sequences are indicated by red and blue colors, respectively.

Based on the established BQQ–OP reactivity and specificity for triplex DNA [11,13], we hypothesized that the observed VA RNAI cleavage by the BQQ–OP (Figure 1B) indicates a potential triplex structure formation within the HAdV-4 VA RNAI molecule. DNA–DNA or DNA–RNA triplex structures are formed by the hydrogen-bonding between the Watson–Crick base-paired polypurine/polypyrimidine double-strand sequences and either a pyrimidine-rich or a purine-rich third strand via Hoogsteen or reverse Hoogsteen base-pairing [4,34]. The closer inspection of the HAdV-4 VA RNAI structure suggested that the three G-C base pairs in the conserved tetranucleotide motif 5′-GGGU-3′ in stem 4 might form a triplex via Hoogsteen base-pairing with three consecutive C nucleotides present in the VA RNAI stem 7 (Figure 2A,B). In order to experimentally confirm the involvement of the 5′-GGGU-3′ sequence motif in stem 4 and the consecutive C nucleotides in stem 7 in the potential RNA triplex structure formation, two HAdV-4 VA-RNAI mutants were constructed. First, one of the G-C base pairs in the tetranucleotide 5′-GGGU-3′ sequence motif was inverted to a C-G base pair, resulting in a perfect stem structure with the same GC content as the wild-type VA RNAI (Figure 2C,D). We predicted that this mutation prevents the VA RNAI triplex formation due to the disruption of the continuous polypurine/polypyrimidine stretch. The second HAdV-4 VA RNAI mutant was created by exchanging the three consecutive C nucleotides in stem 7 with a 5′-AUG-3′ sequence (Figure 2E). Similar to the VA RNAI stem 4 mutant (hereafter as S4M), the stem 7 mutant (hereafter as S7M) was predicted to be deficient in its ability to form triplex structures. Both mutants and the wild-type VA-RNAI (hereafter as Wt) were tested for BQQ–OP reactivity. Again, a specific cleavage at the nucleotide G36 was detected in the case of the VA RNAI (Wt), whereas no specific cleavage was observed in the VA RNAI (S4M) (Figure 2C,D). These results suggest that the conserved 5′-GGGU-3′ motif in the VA RNAI is engaged in the potential triplex formation and that disruption of the integrity of this sequence motif prevented the formation of a stable triplex structure, as shown by the lack of VA RNAI cleavage by BQQ–OP. Surprisingly, the VA RNAI (S7M) was still partially cleaved in the presence of BQQ–OP (Figure 2E). However, a closer inspection of the cleavage products revealed that BQQ–OP cleaved the VA RNAI (S7M) not at the expected nucleotide G36, but at the nucleotide G29. These results suggest that disruption of the three consecutive C nucleotides in stem 7 affects the HAdV-4 VA RNAI potential triplex structure formation at the conserved tetranucleotide motif, 5′-GGGU-3′. However, in contrast to the VA RNAI (S4M), a potential triplex, although different from the one shown in the VA RNAI (Wt), is still formed in the VA RNAI (S7M), as demonstrated by the BQQ–OP cleavage at the nucleotide G29. This observation might indicate that the substitution of the three consecutive nucleotides in stem 7 alters the VA RNAI structure, making the potential RNA triplex formation at the 5′-GGGU-3′ motif unfeasible. Collectively, the BQQ–OP in vitro cleavage experiments demonstrate that particular point mutations in the HAdV-4 VA RNAI sequences can have a detectable impact on the VA RNAI tertiary structure.

### 2.3. HAdV-4 VA RNAI (S7M) Is Partially Deficient in Complementing VA RNAI Lacking Virus Replication

**Figure 3:**
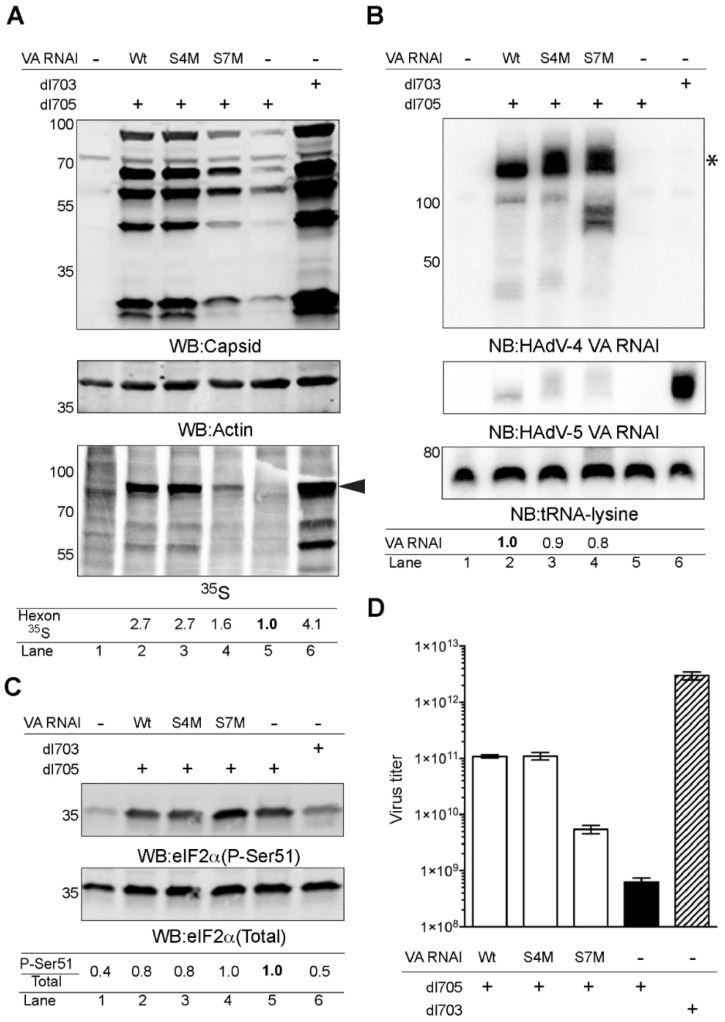
HAdV-4 VA RNAI (S7M) is partially deficient in complementing VA RNAI-lacking virus (dl705) growth. (**A**) Virus capsid protein production in dl705 virus-infected and transient HAdV-4 VA RNAI expressing HEK293 cells. Whole-cell lysates were analyzed by western blotting (WB) and autoradiography (^35^S). The hyphen (-) indicates a sample transfected with a control plasmid, pUC19. The plus (+) indicates a virus-infected sample. The black arrowhead indicates the migration of the virus capsid protein, hexon. Relative synthesis of the ^35^S-labelled hexon protein is shown below the image. (**B**) Ectopic expression of HAdV-4 VA RNAI mutants in HEK293 cells. Total RNA was analyzed by northern blotting (NB) with probes against VA RNAI and tRNA-lysine. Relative HAdV-4 VA RNAI expression from two independent experiments is shown below the image. The asterisk (*) marks the full-length HAdV-4 VA RNAI. (**C**) The same protein lysates as shown in (**A**) were re-analyzed using the anti-eIF2α (Total) and anti-eIF2α (P-Ser51) antibodies. Relative accumulation of the P-Ser51 signal in the total eIF2α protein pool (P-Ser51/Total) from two independent experiments is shown. (**D**) Formation of infectious virus particles in HAdV-4 VA RNAI expressing HEK293 cells. Virus titers were determined in 911 cells after re-infection with HEK293 cell lysates transfected with the indicated plasmids and infected with VA RNAI-deficient dl705 virus. The bars denote the mean virus titer (log10) ± standard deviation of the means. In all of the experiments, wild-type HAdV-5 virus (dl703), expressing both VA RNAI and VA RNAII, was used as the control.

The observation that the above described HAdV-4 VA RNAI mutants altered the BQQ–OP cleavage in vitro (Figure 2) urged us to test the functionality of the VA RNAI (S4M) and VA RNAI (S7M) in vivo. For this purpose, HEK293 cells were transfected with plasmids encoding the HAdV-4 VA RNAI (Wt), VA RNAI (S4M), and VA RNAI (S7M), followed by subsequent infection with the VA RNAI-deficient virus (dl705). Since VA RNAI is essential for virus growth, dl705 is deficient in virus capsid protein synthesis. The ectopic expression of the VA RNAI in dl705-infected cells should revert this deficiency and allow the expression of the virus capsid proteins. Accordingly, monitoring of the capsid protein synthesis and accumulation can be used as a read-out of the virus growth efficiency. The virus capsid protein synthesis and accumulation were monitored by ^35^S pulse-labelling and western blotting, respectively (Figure 3A). In accordance with our previous results [25], the ectopic expression of the HAdV-4 VA RNAI (Wt) enhanced the virus capsid protein production in the dl705-infected cells (Figure 3A, compare lanes 2 and 5). In contrast, the VA RNAI (S7M) was partially deficient in rescuing the capsid protein synthesis (Figure 3A, compare lanes 2, 4, and 5). Interestingly, the VA RNAI (S4M), which was resistant to the BQQ–OP cleavage (Figure 2D), rescued as efficiently as did the VA RNAI (Wt) virus capsid protein synthesis in the dl705 -infected cells (Figure 3A, compare lanes 2, 3, and 5). A northern blot analysis from the same experiment revealed that all three HAdV-4 VA RNAI species were expressed at similar levels in the transfected HEK293 cells. The relative quantification from two independent experiments showed that the VA RNAI (S7M) accumulation was slightly reduced compared to the VA RNAI (Wt). In addition, shorter forms of the VA RNAI (S7M) were detected, suggesting that mutations in stem 7 make the VA RNAI slightly more prone to degradation in HEK293 cells (Figure 3B).

The previous studies have shown that VA RNAI stimulates the HAdV capsid protein synthesis by suppressing eIF2α serine 51 phosphorylation (P-Ser51) (reviewed in [21,22]). In order to test if the described HAdV-4 VA RNAI mutants influence eIF2α phosphorylation, the samples shown in Figure 3A were re-run, and a western blot membrane was probed with the antibodies recognizing phosphorylated eIF2α (P-Ser51) and total eIF2α, respectively. The quantification of two independent experiments showed that both the VA RNAI (Wt) and VA RNAI (S4M) diminished the P-Ser51 signal (Figure 3C, compare lanes 2, 3, and 5). In contrast, the VA RNAI (S7M) expression did not affect the eIF2α phosphorylation in the dl705 virus-infected cells (Figure 3C, compare lanes 4 to 5). Notably, changes in the HAdV capsid protein synthesis do not necessarily correlate with the formation of infectious virus particles [35]. Therefore, we tested how the ectopic expression of the HAdV-4 VA RNAI (S4M) and VA RNAI (S7M) in HEK293 cells influence the formation of the infectious dl705 virus progeny. In accordance with the virus capsid protein accumulation patterns (Figure 3A), the expression of the VA RNAI (Wt) and VA RNAI (S4M) stimulated infectious dl705 virus production, whereas the VA RNAI (S7M) expression resulted in the reduced production of the infectious dl705 virus (Figure 3D).

## 3. Discussion

In this report, we aim to analyze the potential formation of higher-order structures among different HAdV VA RNAI molecules. Due to our previous successful experiences with the BQQ–OP as a DNA triplex-specific probing reagent [11,12,13,14], we applied the same assay to probe for the potential RNA triplex formation in different VA RNAIs. Our results indicate that the BQQ–OP causes specific VA RNAI cleavage, which could be due to the possible formation of an intramolecular triplex structure within the VA RNAI molecule. The cleavage pattern showed considerable variations between different VA RNAI originating from the HAdV-4, HAdV-37, HAdV-5, and HAdV-12. Particularly, the HAdV-4 VA RNAI was cleaved in the presence of BQQ–OP, whereas the HAdV-12 VA RNAI was clearly resistant to the same BQQ–OP treatment (Figure 1). As the BQQ–OP can recognize and induce the cleavage of nucleic acid triplex structures, we favor a view whereby the HAdV-4 VA RNAI adopts a short triplex structure involving the interactions between the stem 4 and stem 7 sequences (Figure 2B). This is supported by the proposed secondary structures of the HAdV-4 and HAdV-12 VA RNAI [28]. Notably, the HAdV-12 VA RNAI lacks three consecutive C nucleotides present in the HAdV-4 VA RNAI stem 7 (Figure 1B,E), which would affect a triplex formation at the conserved tetranucleotide motif, 5′-GGGU-3′. The observed differences in the BQQ–OP reactivity (Figure 1) and, consequently, the probable variations in the RNA higher-order structures can explain our previous results where the HAdV-4 VA RNAI was found to be more effective compared to its HAdV-12 counterpart in rescuing the dl705 virus growth [25]. In addition, Wu and his colleagues have shown that the HAdV-12 VA RNAI fails to block PKR activation, resulting in the prolonged phosphorylation of eIF2α, low translational efficiency of the virus capsid proteins, and the accelerated death of the infected A549 cells [36]. Thus, it is possible that the low virulence of the HAdV-12 in human tissue culture cells is because its encoded VA RNAI does not adopt the higher-order RNA structures, such as triplex, needed to block the anti-viral dsRNA-activated kinase PKR.

Based on our in vitro BQQ–OP cleavage experiments, we hypothesized that the tetranucleotide motif, 5′-GGGU-3′, in stem 4, and the three consecutive C nucleotides in stem 7, are important for the HAdV-4 VA RNAI higher-order triplex structure formation and, consequently, to its in vivo function. However, the in vitro and in vivo experiments with the HAdV-4 VA RNAI S4M and S7M mutants were somewhat conflicting (Figure 2 and Figure 3). This is not entirely surprising, as a discrepancy between the RNA triplex function assessed in the in vitro and in vivo experimental systems has also been reported previously [5]. One potential reason why VA RNAI (S4M) was fully functional in vivo is that the mutated motif, 5′-GGGU-3′, in the VA RNAI (S4M) can form two non-consecutive C*G-C base triads (Figure 2B), which, in turn, contribute to the overall VA RNAI stability. Therefore, the VA RNAI (S4M) may still form a proper higher-order structure in vivo and behave as the VA RNAI (Wt) does in the infected cells. In contrast, the same single inversion mutation in the VA RNAI (S4M) is enough to block the formation of the triplex structure in vitro, as the two remaining non-consecutive C*G-C base triads (Figure 2B) would not be able to accommodate the BQQ–OP intercalating compound and, as a consequence, the cleavage of the VA RNAI (S4M) will not be detected (Figure 2D).

In the case of the VA RNAI (S7M), the substituted three consecutive C nucleotides inhibit the formation of a potential triplex structure at the conserved tetranucleotide motif, 5′-GGGU-3′, in vitro (Figure 2E) and, also, partially affect the ability of the VA RNAI (S7M) to rescue the VA RNAI-deficient virus growth in vivo (Figure 3D). Interestingly, the VA RNAI (S7M) was more prone to degradation when compared to the VA RNAI (Wt) and VA RNAI (S4M) (Figure 3B). This suggests that alterations in the HAdV-4 VA RNAI structure and, particularly in stem 7, influence the stability of the VA RNAI in the infected cells. Hence, the failure of the VA RNAI (S7M) to fully rescue dl705 growth (Figure 3D) can be due to the reduced VA RNAI (S7M) stability, which does not allow for the full inhibition of the eIF2α phosphorylation (Figure 3C).

Among the tested VA RNAIs, the HAdV-4 VA RNAI seems to adopt a potential triplex structure (Figure 1 and Figure 2). Additionally, it blocks the eIF2α(Ser51) phosphorylation most efficiently and rescues dl705 virus growth [25]. Since the HAdV-4 causes epidemic outbreaks of acute respiratory illnesses [18], it is possible that the high pathogenicity of the HAdV-4 relies partially on its encoded VA RNAI. Notably, the HAdV-4 VA RNAI has not been studied in the same detail as the prototypical HAdV-2 and HAdV-5 VA RNAI [30,31,32]. Hence, the HAdV-4 VA RNAI crystal structure, small-angle X-ray scattering measurements, and additional chemical probing are needed to reveal the structural details of this potentially highly pathogenic non-coding RNA molecule.

## 4. Materials and Methods

### 4.1. Plasmids and In Vitro Transcription Reaction

The plasmids encoding HAdV-4, HAdV-5, HAdV-12, and HAdV-37 VA RNAI have been described previously [25]. HAdV-4 VA RNAI stem 4 mutant (S4M) and stem 7 mutant (S7M) sequences (Supplementary Table S1) were synthesized as gBlocks^®^ Gene Fragments (IDT) and cloned into pUC19 plasmid using EcoRI and XbaI restriction enzymes. All VA RNAI sequences were PCR-amplified using a forward primer containing the T7 promoter sequence (Supplementary Table S2). To enhance VA RNAI yields in the T7 polymerase transcription reaction, the VA RNAI transcription start site (positions +1 to +3) was designed to contain the 5′-GGG-3′ sequence. Purified PCR products were used as the templates for the in vitro transcription reaction with a TranscriptAid T7 High Yield Transcription kit (Thermo Scientific, Waltham, MA, USA).

### 4.2. 3′-End Labelling of VA RNAI

One pmol of in vitro transcribed that the VA RNAI was 3′-end labelled using 10 μCi PcP [5′P^32^] (Perklin Elmer, Hopkinton, MA, USA) and T4 RNA ligase (NEB, Ipswich, MA, USA) at 37 °C for 1 h. Radioactively labelled VA RNAI was purified using G-25 column (GE Healthcare, Chicago, IL, USA) and diluted in H_2_O to a final concentration of 100 nM.

### 4.3. Sanger Sequencing

Sanger sequencing reactions on VA RNAI encoding plasmids, using 5′-end radioactively labelled primers (Supplementary Table S2), were used to generate a VA RNAI single nucleotide sequence marker. DNA oligonucleotides were 5′-end labelled using [γ-^32^P] ATP and T4 polynucleotide kinase (NEB) followed by purification on the G-25 column (GE Healthcare). The Sanger sequencing was performed using a USB^®^ Thermo Sequenase Cycle Sequencing Kit (Affymetrix, Santa Clara, CA, USA) according to the manufacturer’s protocol.

### 4.4. BQQ–OP Triplex-Specific Cleavage

BQQ–OP was synthesized, as described previously [8]. Fifty-five nM of VA RNAI (5 nM of ^32^P-labelled VA RNAI + 50 nM of unlabelled VA RNAI) was incubated in the reaction buffer (10 mM Tris-HCl, pH 7.5, 100 mM NaCl, 2 mM MgCl_2_) at 4 °C for 2 h. BQQ–OP (1 μM) and CuSO_4_ (1.5 μM) were premixed and incubated at room temperature for 15 min [8]. Thereafter, the VA RNAI was added to the BQQ–OP/CuSO_4_ reaction mixture, and the sample was incubated at room temperature for 45 min. The BQQ–OP cleavage of the VA RNAI was induced by the addition of mercaptopropionic acid (2 mM, Sigma, St. Louis, MO, USA) to the reaction mixture. The cleavage reaction was performed at room temperature for 3 h. The reaction was stopped by an addition of 2× denaturing RNA loading dye (90% formamide, 0.5 mM EDTA, 0.1% xylene cyanol, 0.1% bromophenol blue). The samples were heated at 95 °C for 5 min and snap-cooled on ice. All samples were analyzed using 8% denaturing (7M urea) polyacrylamide gel electrophoresis in a 1× TBE buffer at room temperature for 3 h. The gel was exposed on PhosphoImager screens, scanned using a Pharos FX^TM^ Plus Molecular Imager (Bio-Rad, Hercules, CA, USA), and analysed using a Quantity One 4.6.9 (Bio-Rad).

### 4.5. Cell Culture, Transient Transfection, Virus Infection, and Virus Titration

Human embryonic kidney cell line, 293 (HEK293), and human embryonic retina cell line, 911 (911), were grown in Dulbecco’s modified Eagle medium (DMEM, Invitrogen, Waltham, MA, USA) and supplemented with 10% fetal calf serum (FCS, PAA, Pasching, Austria), 100 U/mL penicillin, and 100 U/mL streptomycin (PEST, Gibco, Grand Island, NY, USA). HEK293 cells grown on 60 mm plates were transfected using a Turbofect transfection reagent (Thermo Scientific). The cells were infected (5 FFU/per cell) 24 h post-transfection (hpt) with the HAdV-5 virus, dl705, which is deficient in VA RNAI expression [23]. The wild-type HAdV-5 virus (dl703), expressing both VA RNAI and VA RNAII, was used as the control. Virus titration experiments in 911 (Figure 3D) and endpoint virus titer calculations were done, as previously described [25].

### 4.6. Metabolic Labelling of Proteins and Western Blot

Both procedures have been described in detail in our previous publication [25]. The following primary antibodies were used for western blotting: anti-HAdV capsid (1:5000; Abcam, Cambridge, UK, ab6982), anti-actin (1:1000; Santa Cruz, sc-1616), anti-eIF2 (1:500, Abcam, ab26197), and anti-eIF2α(P-Ser51) (1:500, Abcam, ab32157). The ^35^S-signals were detected with Pharos FX^TM^ Plus Molecular Imager and analysed using Quantity One 4.6.9 (Bio-Rad).

### 4.7. Total RNA Isolation and Northern Blot

Total RNA was extracted using the TRIreagent (Sigma), as previously described [37]. Ten micrograms of total RNA were separated on a denaturing 12% polyacrylamide gel (8M urea, 1 × MOPS buffer) and transferred to a Hybond NX membrane (Amersham Biosciences, Piscataway, NJ, USA) with semidry electroblotting [38]. RNA was crosslinked (1200 × 100 μJ/cm^2^, 254 nm) to the membrane using a CL-1000 UV Crosslinker (UVP, CA, USA). The membrane was hybridized in an ULTRAhyb-Oligo buffer (Thermo Fisher, Waltham, MA, USA) with γ-^32^P-ATP labelled hybridization probes detecting 5′ and 3′ ends of the HAdV-4 VA RNAI, HAdV-5 VA RNAI, or tRNA-Lysine (Supplementary Table S2). After overnight hybridization, the membrane was washed 3× for 10 min at 42 °C in a 3× SSC, 0.5% SDS buffer followed by a single wash with a 1× SSC, 0.5% SDS buffer for 15 min at 42 °C. Radioactive signals were detected using a Pharos FX^TM^ Plus Molecular Imager and analysed using a Quantity One 4.6.9 (Bio-Rad).

## 5. Conclusions

Taken together, we propose that the pathogenic HAdV-4 VA RNAI may form a triplex structure, involving the highly conserved stem 4 of the central domain and side stem 7. Our experiments with VA RNAI indicate that BQQ–OP has the potential to be used as an RNA triplex structure probing agent. However, it remains to be tested whether BQQ–OP can be applied to study other triplex-forming RNA molecules.

## Supplementary Materials

The following supporting information can be downloaded at: https://www.mdpi.com/article/10.3390/ijms23063103/s1.
