# Supplementary material for: Structural Insights into Human Adenovirus Type 4 Virus-Associated RNA I
Source: Int J Mol Sci. 2022 Mar 13;23(6):3103. doi: 10.3390/ijms23063103 (PMC8949993; doi:10.3390/ijms23063103)
Supplement: Supplementary file 1 [file ijms-23-03103-s001.zip › ijms-1610197-supplementary.pdf]

Supplementary Table S1. HAdV VA RNAI sequences.

VA RNAI sequences	
HAdV-4	5′GGGCUCGACUCCGUGGCCUGGAGGCUAAGCGAACGGGUUGGGCUGCGCGUGUACC<br>CCGGUUCGAAUCUCGAAUCAGGCUGGAGCCGCAGCUAACGUGGUACUGGCACUCCCG<br>UCUCGACCCAGGCCUGCACAAAACCUCCAGGAUACGGAGGCGGGUCGUUUU-3′
HAdV-4 S7M	5′GGGCUCGACUCCGUGGCCUGGAGGCUAAGCGAACGCGUUGGGCUGCGCGUGUACC<br>CCGGUUCGAAUCUCGAAUCAGGCUGGAGCCGCAGCUAACCUATUACUGGCACUATGG<br>UCUCGACGCAGGCCUGCACAAAACCUCCAGGAUACGGAGGCGGGUCGUUUU-3′
HAdV-4 S4M	5′GGGCUCGACUCCGUGGCCUGGAGGCUAAGCGAACGCGUUGGGCUGCGCGUGUACC<br>CCGGUUCGAAUCUCGAAUCAGGCUGGAGCCGCAGCUAACGUGGUACUGGCACUCCCG<br>UCUCGACGCAGGCCUGCACAAAACCUCCAGGAUACGGAGGCGGGUCGUUUU-3′
HAdV-5	5′GGGCACUCUUCCGUGGUCUGGUGGAUAAAUUCGCAAGGGUAUCAUGGCGGACGAC<br>CGGGGUUCGAGCCCCGUAUCCGGCCGUCCGCCGUGAUCCAUGCGGUUACCGCCCGCG<br>UGUCGAACCCAGGUGUGCGACGUCAGACAACGGGGGAGUGCUCCUUUU-3′
HAdV-12	5′GGGACUCCCUUCCGUGGUUUGGUGGAAAAGUCACAAGGGUACCAUAGCGAGGAAC<br>CCCGGUUCGAAACCGGCAGGAUCCGCUAUGAGCACAAGUGAGGCGCUUGCGCGUUGA<br>ACCCGGCCAAGGACCCCCAGACACGGAGAGGAGUCUUUUUUU-3′
HAdV-37	5′GGGCUCUUCCUCCGUAGCCUGGCGGAACGCAAACGGGUUAGGCCGCGUGUGUACC<br>CCGGUUCGAGUCCCCUCGAAUCAGGCUGGAGCCGCGACUAACGUGGUAUUGGCACUC<br>CCGUCUCGACCCAAGCCCGAUAGCCGCCAGGAUACGGCGGAGAGCCCUUUUU-3′

Red = mutation in Stem 4 (S4M), Blue = mutation in Stem 7 (S7M).

Supplementary Table S2. Primers used for PCR and Northern blot.

**PCR primers to generate VA RNAI templates for *in vitro* transcription**		
Target		Sequence 5'-3'
HAdV-4	F-Primer	GAAAT**TAATACGACTCACTATA**GGGCTCGACTCCGTGGCC
	R-Primer*	**AAAACGACCCGCCTCCGTATCC**
HAdV-5	F-Primer	GAAAT**TAATACGACTCACTATA**GGGCACTCTTCCGTGGTC
	R-Primer *	**AAAAGGAGCACTCCCCCG**
HAdV-12	F-Primer	GAAAT**TAATACGACTCACTATA**GGGACTCCCTTCCGTGG
	R-Primer*	**AAAAGACTCCTCTCCGTG**
HAdV-37	F-Primer	GAAAT**TAATACGACTCACTATA**GGGCTCTTCCTCCGTAGC
	R-Primer*	**AAAAGGGCTCTCCGCCG**
**Primers used as the probes for Northern Blot**		
Target		Sequence 5'-3'
HAdV-4	5'-end	GTTCGCTTAGCCTCCAGGCCACGGAGTCGAGCC
	3'-end	AAAACGACCCGCCTCCGTATCCTGGAGGTTTTG
HAdV-5	5'-end	GAATTTATCCACCAGACCACGGAAGAGTGCC
	3'-end	AAAAGGAGCACTCCCCCGTTGTCTGACGTCGCA
tRNA-Lysine		ACCGACTGAGCTATCCGGGC

Bold = T7 promoter sequence, underlined= VA-RNA sequence, red= extra G added to enhance T7 transcription. F = forward, R = reverse. * = primers used for Sanger sequencing reaction.
